# An Analysis of the Effect of Activation Temperature and Crack Geometry on the Healing Efficiency of Polycaprolactone (PCL)/Epoxy Blends

**DOI:** 10.3390/polym15020336

**Published:** 2023-01-09

**Authors:** Rocío Calderón-Villajos, Xoan Fernández Sánchez-Romate, Alberto Jiménez-Suárez, Silvia González Prolongo

**Affiliations:** Materials Science and Engineering Area, Escuela Superior de Ciencias Experimentales y Tecnología, Universidad Rey Juan Carlos, Calle Tulipán s/n, Móstoles, 28933 Madrid, Spain

**Keywords:** self-healing, epoxy blend, polycaprolactone

## Abstract

Self-healing materials have attracted great interest in recent years. Particularly, the use of thermoset/thermoplastics blends has emerged as a good option with relatively low activation temperatures and potential infinite healing cycles. Nevertheless, a methodical study of healing conditions and evaluation is still required for further industrial development. The effect of activation temperature and crack morphology in polycaprolactone (PCL)/epoxy blends are explored. For this purpose, PCL content was varied (5, 10, and 15 wt %) with contents lower than critical composition. Therefore, the morphology of all studied blends is the epoxy matrix with a separated PCL phase. In this sense, an increase in PCL content leads to a reduction in the Tg, due to the partial PCL miscibility, and the presence of larger PCL domains. It was observed that a higher temperature (150 °C) and PCL content led to a more efficient self-healing process because of both the lower viscosity of the melted PCL at higher temperatures and the presence of larger PCL reservoirs when increasing the PCL content. Crack morphology influence was studied by inducing cracks with different tools: a custom crack machine with a cutting blade, a scalpel, and a pin. The results show that the recovery was better when the cracks were smaller and shallower, that is, with the pin. In addition, the healing efficiency by means of both parameters, crack volume and depth change, showed more similar results in slimmer cracks, due to a lower crack width-to-depth ratio.

## 1. Introduction

Some industries have a growing interest in removing corrective maintenance to avoid additional operational costs. In this regard, it is necessary to explore new materials with novel functionalities. Among these functionalities, self-healing is of interest [1], as it would allow for restoring the initial properties via an autonomous or external stimulus, without the need for patches or other repairs. However, the most common polymer matrices used in the aircraft or automotive industries are thermosets [2,3], which do not present any self-healing properties due to the irreversibility of curing reaction that impedes reprocessing or welding. Therefore, it is important to explore other ways of promoting self-healing capabilities [4,5].

The reason for the use of thermosets lies in the fact that they have excellent engineering properties due to their thermal and chemical resistance, which make them an excellent candidate/choice for industrial applications [6]. In addition, structural thermosets, such as epoxy matrices, present good mechanical properties in terms of strength and stiffness in comparison with thermoplastics [7]. However, their low fracture toughness and strain at break can lead to the appearance of premature defects that can quickly propagate until catastrophic failure. Therefore, the need for self-healing capabilities is more prevalent in this type of materials [8,9].

There are several mechanisms to introduce self-healing abilities in thermosetting polymers: incorporating microcapsules containing the healing agent, which is released where a crack propagates and breaks the microcapsules [10]; modifying their network formulation by creating covalent adaptable networks (CAN) [11] based on the introduction of reversible bonds; or adding thermoplastic phases in the form of blends [12], which allows for thermoplastic flow through a crack when increasing the temperature above its melting point [4]. Apart from the self-healing properties, it is also of high interest to investigate the effect of the modification of the polymer network via microcapsules [13], CAN [14], the reversibility of non-covalent bonding in supramolecular thermosets [15], or via the addition of a thermoplastic phase on the mechanical performance of the system [16,17]. In this latter area, PCL is a good candidate as a thermoplastic phase [18] in epoxy systems as it both improves the fracture toughness of the original matrix and promotes the self-healing abilities of the resulting blend [12,19]. In fact, the addition of PCL and other thermoplastic phases has proved to have excellent capabilities to enhance interlaminar and impact properties in fiber-reinforced composites, although a reduction in the glass transition temperature can be also observed [20,21]. Other approaches such as supramolecular thermosets can avoid this effect of Tg decrease as they do not incorporate a second phase; nevertheless, they are usually activated by other external stimuli rather than temperature, such as pH [15]. Moreover, the selection of PCL as the thermoplastic phase is explained by the PCL expansion when melted, which is not present in other semicrystalline thermoplastics [22].

These synergistic effects are explained because PCL is miscible in the epoxy monomer. However, under certain conditions (cloud point), there is a phase separation during curing which leads to a restriction of the toughening phase and a better interaction with the epoxy matrix [23,24]. Therefore, the self-healing process is triggered by heating the blend above the melting point of PCL (which is around 60 °C) [25], so that the PCL flows in a molten state into the cracks and fills them up. It has been observed in previous studies that the self-healing capabilities achieved are quite good in PCL/epoxy blends and mainly depend on the PCL content, phase separation, morphology, and distribution, which in turn depend on the PCL content for a fixed monomer/hardener system and its curing cycle [18,26,27].

However, the effect of the activation temperature and the crack geometry remains to be investigated. For this reason, this research aims to explore how these parameters affect the self-healing process. First, the effect of activation temperature is investigated, and then, once this temperature is optimized, the effect of crack geometry is explored.

To achieve this purpose, the conditions that influence the geometry of the shape were carefully controlled. Different tools were used to induce the damage in the blends with different morphologies: a custom crack machine with a cutting blade, a scalpel, and a pin. A study of the impact of the crack geometry on the self-healing process helps to identify the efficiency against different types of defects or damage.

Several previous research efforts focus on qualitative self-healing results using micrographs or photography [18,27,28,29,30,31] without providing a standard means of quantifying the recovery. This makes the comparison among different materials or techniques difficult, which may prevent potential users of this technology to invest in it due to the uncertainties about the benefit.

This paper proposes two quantitative methods to measure the amount of self-recovery by comparing the volume change in terms of percentage, *V*%, and by comparing the depth change, µ%, before and after the self-healing process. These measurements are taken by using a software package so that they can be easily reproduced. The results would shed light on the influence of these parameters on the healing process in PCL/epoxy blends and establish a rigorous and reproducible method to evaluate self-healing capabilities that would allow future comparison among different research.

## 2. Experimental Procedure 

### 2.1. Materials

The epoxy resin was based on diglycidyl ether of bisphenol A (DGEBA) with a molecular weight of 340.41 g/mol and a density of 1.16 g/cm^3^ cured with the crosslinker 4,4′ diaminodiphenyl sulfone (DDS). Both were supplied by Sigma Aldrich (Sigma Aldrich, Merck Life Science S.L.U., Madrid, Spain). The thermoplastic polymer used was polycaprolactone (PCL), also supplied by Sigma Aldrich. It had a density of 1.146 g/cm^3^ at 25 °C, a molecular weight of 14,000 g/mol and a melting point of 60 °C. The information was supplied by the manufacturer.

### 2.2. Manufacturing of PCL/Epoxy Blend

As an initial step, the PCL pellets were milled to reduce their initial size and improve their dissolution in the epoxy monomer. A DGEBA monomer was heated to 80 °C and mixed with the desired proportion of fine PCL powder by means of magnetic stirring. This mixture was then magnetically stirred and degassed under vacuum for 15 min at the same temperature. After this, the mixture was heated up to 210 °C, and the DDS crosslinker was added in a stoichiometric proportion of 3.5:1 in mass by means of magnetic stirring to ensure the homogenization of the mixture for 3 min.

The resulting mixture was finally poured into preheated molds and cured at 210 °C for 3 h as this curing cycle maximizes the mechanical properties of the resulting system [32]. Finally, the cured samples were demolded and machined to the dimensions required. Using this process, PCL/epoxy blends with 5, 10, and 15 wt. % of PCL were manufactured to analyze the effect of PCL content on the self-healing properties. These compositions are based on previous research studies, as they allow for reaching good phase separation of PCL in epoxy phase or co-continuous morphology [22].

### 2.3. Dynamic Mechanical Thermal Analysis (DMTA)

The loss modulus and storage modulus were evaluated with DMTA using a DMA QB00 V7.1 module from TA Instruments (TA Instruments –Waters Cromatografía, S.A, Madrid, Spain) at temperatures ranging from 25 to 275 °C and at a heating rate of 2 °C/min. For this purpose, two samples of 37.5 × 12 × 1.5 mm^3^ were tested at single cantilever clamp at 1 Hz frequency and a wave amplitude of 1 % of the thickness of the specimen. In this way, the glass transition temperature, Tg, was set as the maximum of tan δ curve, that is, at the maximum loss modulus to the storage modulus ratio, while the stiffness of the samples was determined through the storage modulus (*E*′) in the glassy stage at 30 °C.

### 2.4. Microstructural Characterization

A microstructural analysis of the PCL/epoxy system was carried out through field emission gun scanning electron microscopy (FEG-SEM) using a Nova NanoSEM FEI 230 apparatus from Philips, installed at Centro de Apoyo Tecnológico in Universidad Rey Juan Carlos, Madrid, Spain. The fracture surfaces under cryogenic conditions were observed to analyze the PCL domains. They were previously coated with a sputtered platinum (Pt) layer of 3.1 nm for proper observation.

### 2.5. Self-Healing Test

The manufactured specimens were subject to several induced damages to analyze the effect of crack geometry on the self-healing capabilities of the materials. These damages were initially caused by means of a custom machine with a blade and a control system that ensures consistent crack depth. After some self-healing tests, two extra methods were used to induce the damage (a scalpel and a pin), so that the impact of the size and shape of the crack could be studied.

After being damaged by the cutting blade machine, the samples were heated with the aim of triggering the self-healing process. The heating time was fixed to 15 min, but four different temperature thresholds were tested: 90 °C, 110 °C, 130 °C, and 150 °C. Initial tests confirmed that the heating time was enough to reach the maximum self-healing efficiency and, on the other hand, that as the temperature rose, self-healing improved. Therefore, the samples damaged with the scalpel and the pin were only tested under 150 °C.

The first method used to quantify the self-healing process consisted in the comparison of 3D micrographs before and after causing the cracks. For this task, the self-healing efficiency by means of volumetric change (*V*%) was calculated using a 3D optical profiler from Zeta Instruments, Zeta-20 model (Zeta Instruments is part of KLA Corporation, Milpitas, CA, USA and the Mountain Map Premium 7.1 software. The volumetric change is defined as follows, Equation (1):

(1) $$V\;\left(\%\right)=\frac{V_{o}-V_{f}}{V_{o}}\times 100$$

where *V_o_* and *V_f_* are the volumes before and after the self-healing process, respectively. To make accurate calculations, the crack perimeter was delimited through the software, which subsequently provides the volume in both cases.

**Figure 1:**
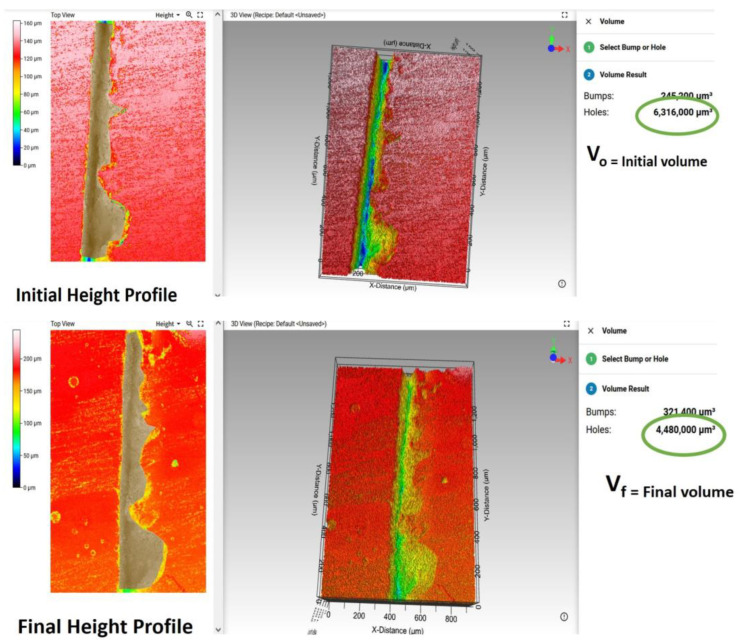
Example of *V_o_* and *V_f_* calculations using Mountain Map Premium 7.1 software. They show how the crack is delimited before and after the self-healing process, and the values provided by the software in both cases.

Figure 1 shows an example of how *V_o_* and *V_f_* were calculated using the software. The measurements were performed in three different samples, and three cracks were made on each of them. The average *V*% was then calculated.

The second method consisted in measuring the average depth before and after the self-healing process, using the same 3D optical profiler. The depth change (µ%) is defined in Equation (2):

(2) $$µ\;\left(\%\right)=\frac{{µ}_{o}-{µ}_{f}}{{µ}_{o}}\times 100$$

where µ*_o_* and µ*_f_* are the depths before and after the self-healing process, respectively.

**Figure 2:**
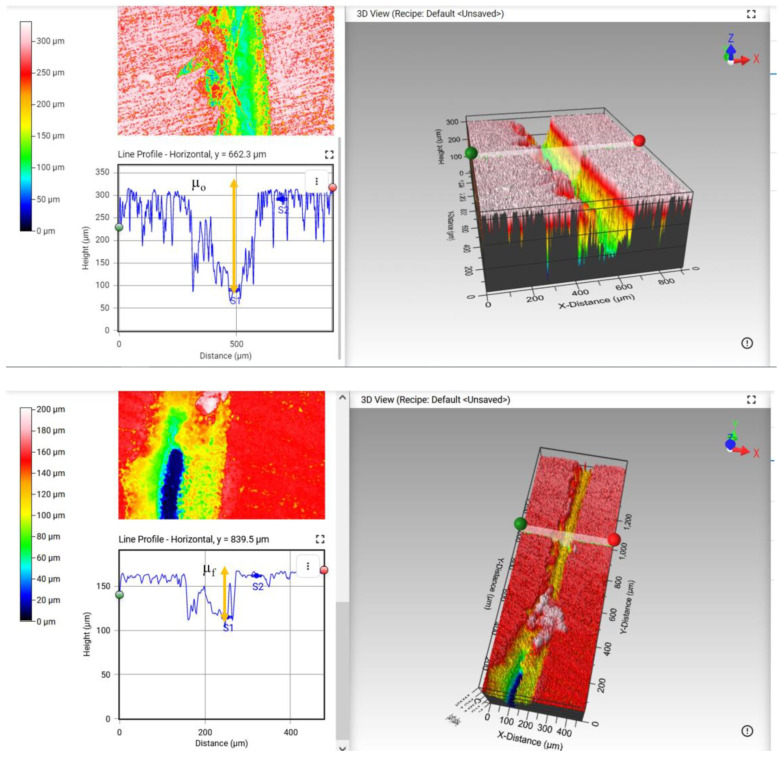
Depth calculations using Mountain Map Premium 7.1 software before and after the self-healing process. After selecting a region of the crack, the program calculates the difference between the highest and lowest point and provides the depth of the crack.

Figure 2 shows how µ*_o_* and µ*_f_* are calculated using the software, which provides the depth difference between the highest and deepest points. Ten measurements were performed on each of the three different samples and then averaged.

## 3. Results

In this section, first, a basic characterization of the studied blend is presented to evaluate the main thermal and mechanical properties of this system. Then, a study of self-healing capabilities as a function of PCL content and triggering temperature is carried out. Then, the effect of crack morphology by using different tools is explored to better understand the self-healing process in PCL/epoxy blends. 

### 3.1. Thermomechanical Analysis of PCL/Epoxy Blend

**Figure 3:**
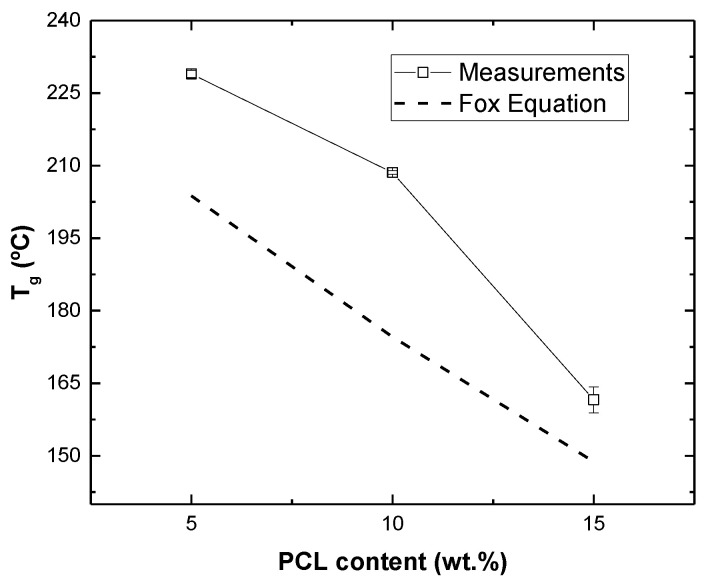
Tg values as a function of PCL content determined experimentally and by using Equation (3).

Figure 3 summarizes the Tg values of the PCL/epoxy system. It can be observed that Tg decreases with PCL content, as expected, due to the partial solubilization of the thermoplastic polymer into the glassy network and the low glass transition temperature of the PCL (−60 °C) in comparison with that of the neat cured DGEBA/DDS resin (237 °C, as observed previously) [32]. More specifically, the variation in the Tg with PCL percentage can be estimated according to the well-known Fox Equation, which correlates the final Tg of the system with the relative proportion of each polymer [33]:

(3) $$\frac{1}{T_{g}}=\frac{w_{1}}{T_{g1}}+\frac{w_{2}}{T_{g2}}$$

where *w*_1_, *T_g_*_1_, *w*_2_ and *T_g_*_2_ are the weight fractions and glass transition temperatures of each component, respectively, and Tg is the final glass transition temperature of the system.

Here, it can be observed that there are some discrepancies between the measured data (square symbols) and those predicted by Equation (3) (dashed line). This is explained because the PCL is not totally miscible, as noted before and, therefore, the values of the Tg obtained experimentally are slightly above those predicted by the Fox Equation, which supposes total miscibility.

Furthermore, the effect of PCL addition can be also investigated in terms of cross-link density. In this case, the effective cross-link density, *υ_c_*, can be calculated by following the formula proposed by L.W. Hill [34]:

(4) $$\upsilon_{c}=\frac{{E{}^{\prime }}_{R}}{3RT}$$

where *E*′*_R_* is the storage modulus of the rubbery state at a temperature *T* = *T_g_* + 30 K, and *R* is the gas constant.

**Figure 4:**
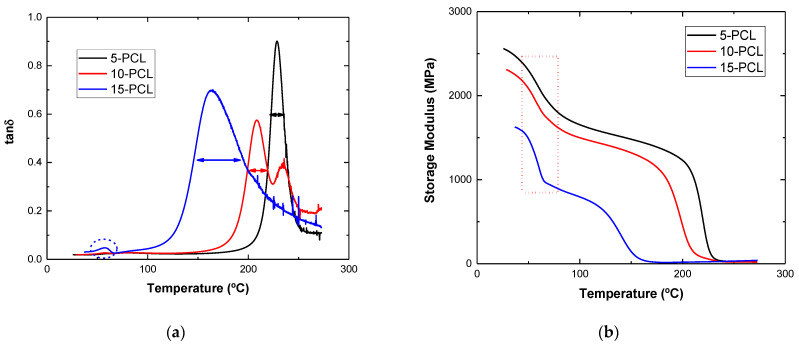
Results of the DMTA tests showing (**a**) the evolution of tanδ and (**b**) the variation in the storage modulus with the temperature for the different conditions.

In this regard, Table 1 shows the values of the cross-link density as a function of PCL content. It can be observed that the PCL increase promotes a reduction in the crosslinking density, meaning that the epoxy is effectively plasticized with the addition of PCL, due to the higher miscibility of this phase in the DGEBA/DDS system [32]. Moreover, this plasticization effect is confirmed through a significant reduction in the storage modulus at 30 °C with the increment of the PCL percentage, as also observed in Table 1 and in Figure 4, changing from 2500 GPa to 1900 GPa for 5 and 15 wt.% PCL samples, respectively. In addition, it can be observed that there is a higher scattering of both crosslinking density and storage modulus values when the amount of PCL increases, especially in the case of 15 wt.% PCL samples. This can be explained by the higher heterogeneity of the polymer network. In fact, it is known that higher amounts of PCL lead to a co-continuous PCL–epoxy network [22,35] and, thus, the characteristics of the polymer network will be significantly affected. This can be confirmed through an analysis of the tanδ curves obtained with DMTA (Figure 4a). Here, the higher PCL contents exhibited a broader tanδ peak (marked with color arrows), implying a broader glass transition region, which is correlated with a higher structural heterogeneity [36].

**Figure 5:**
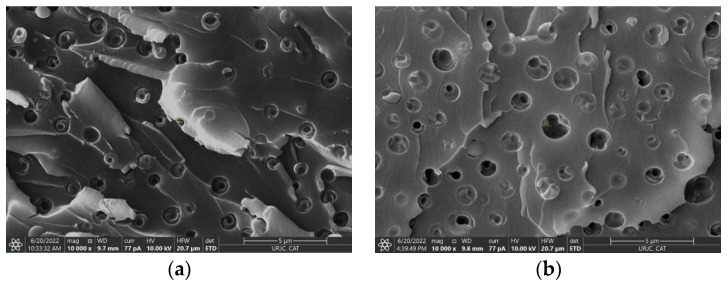

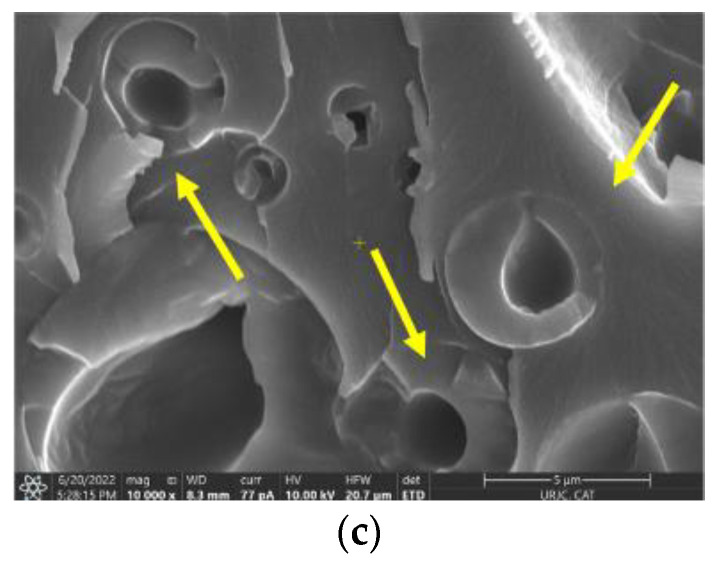
SEM images of the fracture surface of epoxy blends with (**a**) 5, (**b**) 10 and (**c**) 15 wt.% PCL, where the yellow arrows denote the presence of large PCL domains.

Furthermore, the more prevalent effect of PCL on the mechanical properties of the epoxy blend is also confirmed by drastic reduction in the storage modulus at 50–70 °C (Figure 4b), which is the range for the melting temperature of the PCL resin. This drastic reduction is more prevalent in the case of 15 wt.% PCL samples (highlighted regions of Figure 4a,b) and it is explained by the presence of a higher amount of separated, and non-dissolved, PCL phase inside the material. More specifically, according to the SEM images of Figure 5, it can be observed that the average size of the PCL domains is much higher in the case of 15 wt. % PCL samples (Figure 5c) and, therefore, their influence on the mechanical behavior of the system will be much more significant [25].

### 3.2. Self-Healing Ability 

As commented before, the process for triggering the self-healing process in the PCL/epoxy blend consists in heating the samples so that the PCL melts and flows into the cracks to fill them up. In order for this process to occur, the crack needs to go through, or, at least, be close enough to, a PCL reservoir. Therefore, greater self-healing results are expected when increasing the PCL content, since there is a higher probability that the crack would go through a reservoir. However, previous studies of PCL/epoxy blends have shown that raising the percentage of PCL above 15%wt induces a co-continuous phase morphology where the thermomechanical properties of the materials can be decreased, as the PCL presents a lower mechanical strength than neat epoxy resin [22]. Therefore, 15% wt was the maximum amount of PCL used for these experiments. First, the analysis of the self-healing properties will be carried out for the custom crack machine using a cutting blade. Then, to explore the influence of crack geometry, the effect of scalpel and pin cracks will be also explored.

#### 3.2.1. Custom Crack Machine-Induced Damage Analysis

As noted in the Experimental Section, different self-healing activation temperatures of 90, 110, 130 and 150 °C were explored to analyze the influence of the activation temperature on the self-healing ability. In this case, the heating time was set as 15 min to promote a proper PCL flow. 

**Figure 6:**
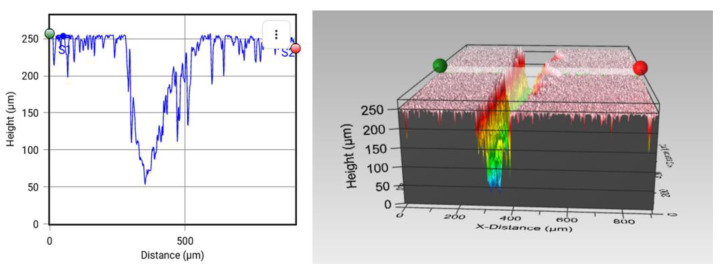
Initial damage created by the custom crack machine.

The first experiment consisted in damaging the samples using the custom crack machine, which created cracks of 270 ± 22 µm of width and 250 ± 20 µm of depth, as observed in Figure 6. The results of the self-healing efficiencies are summarized in Table 2.

It can be observed that the highest self-healing efficiency was just 34% on the sample with 15% wt of PCL at 150 °C. This result was lower than expected, in comparison with previous published results in which healing efficiencies in the range of 50–70% were reached [37]. Furthermore, it can be also noticed that the samples with 5% PCL did not show any healing properties. The reason for this lack of self-healing properties lies in the fact that the crack probably did not go through any PCL reservoir and, therefore, the PCL flow through the cracks was not promoted. As expected, the samples with the intermediate PCL content (10 wt.%) also presented intermediate self-healing properties. Figure S1 shows the images of optical profilometry for 5 and 10 wt.% of the PCL/epoxy blends, where it is possible to perform a qualitative analysis of the self-healing results. As can be seen, samples with 5 wt.% presented less healing than those with 10 wt.%.

**Figure 7:**
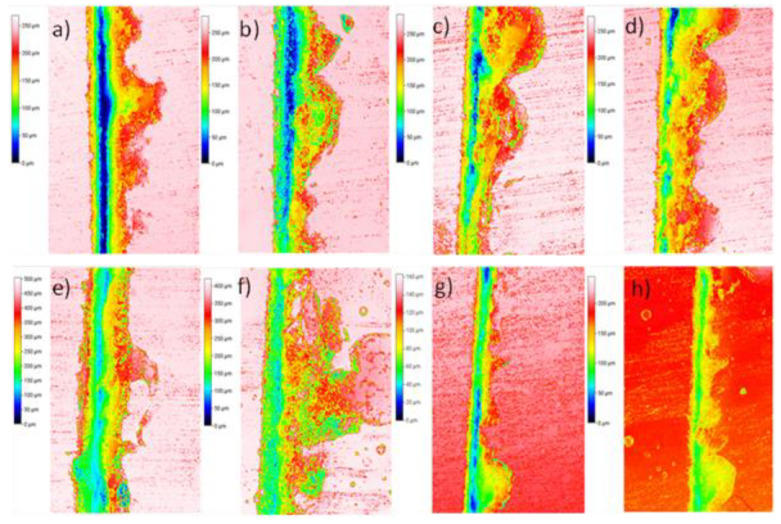
Images of optical profilometry for PCL/epoxy blends with 15% wt PCL before (left image of each pair) and after (right image of each pair) the self-healing process at: (**a**,**b**) 90 °C, (**c**,**d**) 110 °C, (**e**,**f**) 130 °C, (**g**,**h**) 150 °C.

Moreover, the temperature also had a great impact on the self-healing properties. In fact, at 90 °C, no significant self-healing was promoted for any sample. This effect is explained by the higher viscosity of the PCL at lower temperature, where the flowing is not sufficiently promoted and, therefore, the crack is not properly filled. In this sense, the healing efficiencies were increased with temperature, reaching maximum values at 150 °C, where the viscosity of PCL is expected to be low enough for proper crack filling, as shown in Figure 7 for the 15 wt.% samples and in Figure S1 for the 10 and 5 wt.% samples. For this reason, 150 °C should be selected as the optimum temperature for the self-healing process. No further temperatures were explored, as the Tg of the blend with 15 wt.% PCL was around 140 °C (Figure 4) and, thus, above this temperature, the dimensional control would be very difficult.

The reason for the very low healing efficiencies reached can be found in the geometry of the crack. In this case, the dimensions of the crack were very large in comparison with the PCL reservoirs. For this reason, the effect of crack geometry was analyzed. Here, only the 10 and 15 wt.% PCL samples were explored, as the 5 wt.% PCL samples did not show any healing properties, as explained above. The new cracks were created, as mentioned above, by means of a pin and a scalpel for a better damage control.

#### 3.2.2. Scalpel-Induced Damage Analysis

**Figure 8:**
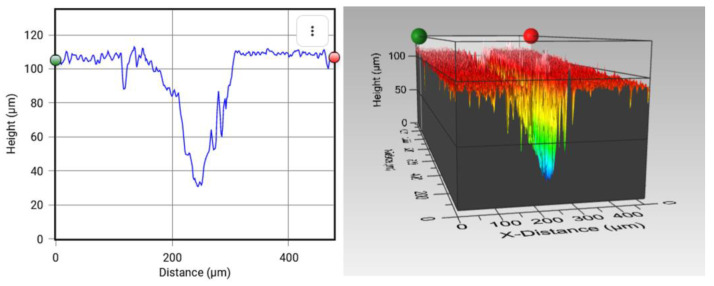
Initial damage created by the scalpel.

The cracks performed with a scalpel presented a lower size of the crack when compared with the custom crack machine as shown at the end of the Section 3.2.3, presenting 80 ± 12 µm of average depth and 100 − 90 ± 12 µm of average width, as shown in Figure 8.

**Figure 9:**
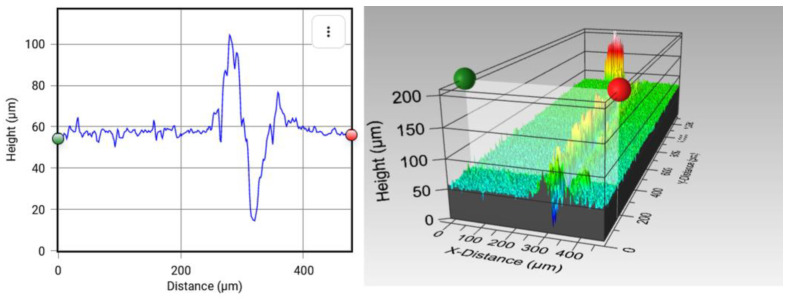
Image of the initial crack using a pin.

**Figure 10:**
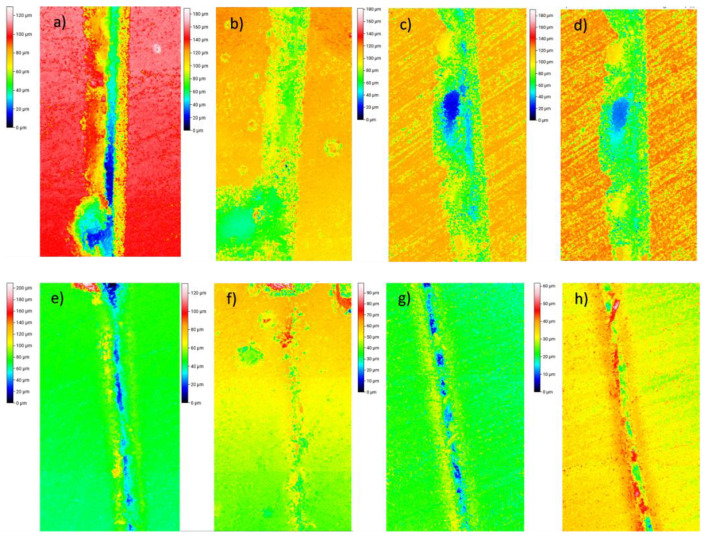
Images of optical profilometry for PCL/epoxy blends: (**a**–**d**) with a 15% and 10% wt PCL before and after the self-healing process at 150 °C, respectively, using a scalpel; (**e**–**h**) with 15% and 10% wt PCL before and after the self-healing process, respectively, using a pin.

The results in Table 3 show that for this type of crack, the self-healing ability was higher than the one generated by the custom crack machine. This is explained by the size of the crack, which is wider and deeper compared to the pin one (Figure 9). Here, the damage induced was significantly lower and, thus, the crack generated could be filled by the PCL reservoirs, once melted, in a more proper way. More specifically, the higher healing efficiency could be stated by analyzing the profilometry images of Figure 10.

**Figure 11:**
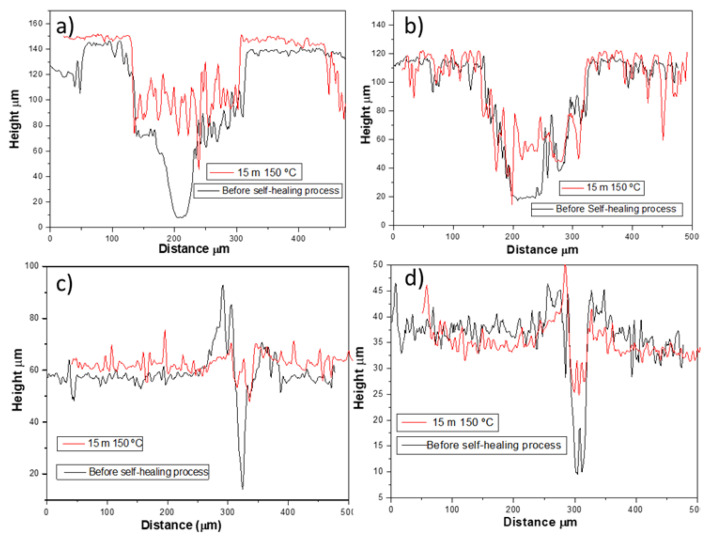
Recovery profile for PCL/epoxy blends: (**a**,**b**) with a 15% and 10% wt PCL before and after the self-healing process at 150 °C, respectively, using a scalpel; (**c**,**d**) with 15% and 10% wt PCL before and after the self-healing process, respectively, using a pin.

Figure 11a,b shows the recovery profiles for PCL/epoxy blends with a 15% and 10% wt PCL before and after the self-healing process. It can be observed that the variations in the crack profiles before and after the self-healing process demonstrate that recovery is occurring.

#### 3.2.3. Pin-Induced Damage Analysis

The last experiment used a pin to produce shallower cracks, as noted before (Figure 11). In this case, the crack width was 40 ± 5 µm and the crack depth 40 ± 5 µm on average, as shown in Figure 9. Here, it can be observed that there was a significant peak around the crack. This can be explained by the effect of a more prevalent plastic deformation in the blend produced by the pin, while the crack was made due to the higher stress concentration in comparison with the custom crack machine and the scalpel, which produced a narrower crack and hence facilitated the rising up of excess material.

As shown in Table 3, the self-healing results for this type of crack were the highest of all experiments, as also confirmed by the profilometry images of Figure 10 and by the recovery profiles of Figure 11c,d.

**Figure 12:**
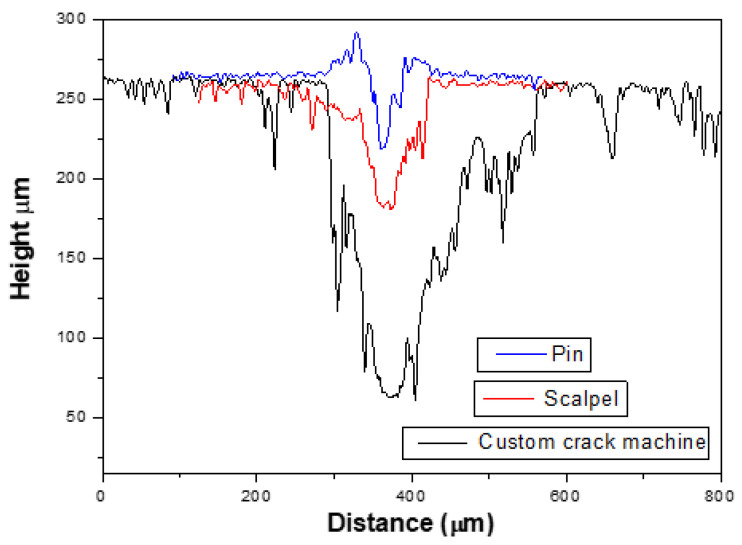
Profile image generated by the three tools used (custom crack machine, scalpel, and pin) in the surface of the PCL/epoxy blends showing the different geometries of the cracks.

The reason for the higher self-healing efficiencies is again based on the crack geometry. In this case, the smallest crack size made possible proper PCL flow during healing activation. In addition, it can be observed that the healing efficiencies by means of μ% and *V*% were quite similar, in a manner opposite to those for the custom crack machine and the scalpel. An explanation may be that the crack geometry was slimmer, that is, the ratio between the crack width and the crack depth was slightly lower. Therefore, the crack reduction after the healing process was comparable in terms of depth change and volume change and, thus, the healing efficiencies reported by these methods were quite similar (see Figure 12).

Furthermore, it can be also elucidated that the samples with the highest amount of PCL showed a higher self-healing efficiency for both scalpel and pin-induced cracks, as expected. Here, it can be also stated that the differences between healing efficiencies of 10 and 15 wt.% PCL samples in the case of the pin were much lower. This can be explained because by the size of the crack. On the one side, the lower crack size induced a much more proper PCL flow through the crack, as noted before, as it was nearer to the size range of the PCL reservoirs. In addition, it should be noted that with such small crack dimensions, a little part of the repair is due to the material’s own plastic deformation [38], which is similar in both cases.

In sum, the results proved that the self-healing efficiency in the PCL/epoxy blend was quite significantly affected by the PCL content, healing temperature, and crack geometry. A better knowledge of the influence of these parameters has been achieved. 

## 4. Conclusions

A study of the self-healing process of the PCL/epoxy blend was carried out. The effects of PCL content, activation temperature, and crack geometry were explored for this purpose.

First, we observed that an increase in PCL contents led to a reduction in the Tg, due to the partial miscibility of the PCL in the epoxy network, as well as to an increase in the size of the PCL domains, due to the phase separation during curing. This explains why the highest self-healing efficiencies were achieved for the 15 wt.% PCL samples, due to the presence of larger PCL reservoirs. In addition, higher temperatures reduced the viscosity of PCL and thereby facilitated the self-healing process, as the PCL could fill the cracks more easily. 

Furthermore, the crack geometry impact was explored using a custom crack machine with a cutting blade, a scalpel, and a pin. We noticed that the narrower and shallower the crack was, the better it was repaired. Therefore, the cracks induced by the pin showed the highest healing efficiencies, whereas the cutting blade presented the lowest one.

The analysis by means of volume change, *V*%, and depth change, μ%, showed that the slimmer the crack was, the more comparable the healing efficiencies of these two methods were. The highest contents of PCL allowed for reaching a volume recovery of 75% when damage was generated with a pin, while it was slightly slower (69%) when using a scalpel. A reduction from 15% to 10% in PCL content led to a volume recovery reduction of 10% and 25% for cracks induced by a pin and scalpel, respectively, highlighting the importance of the PCL content.

Therefore, the results shed light on the manner in which the PCL morphology, activation temperature, and crack geometry affect the healing performance and thus allow for a better knowledge of PCL/epoxy blends. As mentioned before, much research has used different damage generation methodologies and self-healing capability evaluations; thus, the proposed methodology will help to establish a rigorous and comparable methodology.

## Figures and Tables

**Table 1:** Cross-link density (calculated using Equation (4) and glassy storage modulus values (determined at 30 °C) for the different tested conditions.

Condition	$\upsilon_{c}\times 10^{3}$ (mol/cm^3^)	*E′* (MPa)
5-PCL	2030 ± 130	2456 ± 140
10-PCL	1970 ± 420	2344 ± 50
15-PCL	1237 ± 506	1931 ± 435

**Table 2:** Results of the self-healing efficiencies of samples using the custom crack machine with different activation temperatures and PCL concentrations.

	15%		10%		5%	
T (°C)	µ%	*V*%	µ%	*V*%	µ%	*V*%
90	-	-	-	-	-	-
110	13 ± 3	18 ± 4	12 ± 2	22 ± 5	-	-
130	21 ± 2	24 ± 3	18 ± 2	23 ± 6	-	-
150	34 ± 2	38 ± 3	20 ± 1	30 ± 4	-	-

**Table 3:** Results of the self-healing efficiencies of samples damaged with the scalpel and the pin after heating at 150 °C.

% PCL	Scalpel		Pin	
	µ%	*V*%	µ%	*V*%
15	60 ± 7	69 ± 4	72 ± 11	75 ± 10
10	33 ± 8	42 ± 12	66 ± 9	65 ± 13

## Data Availability

Not applicable.

## Supplementary Materials

The following supporting information can be downloaded at: https://www.mdpi.com/article/10.3390/polym15020336/s1, Figure S1: Images of optical profilometry for PCL/epoxy blends with 10% wt PCL before and after the self-healing process after15 minutes of heating at: (**a**,**b**) 110 °C, (**c**,**d**) 130 °C, (**e**,**f**) 150 °C and PCL/epoxy blends with 5% wt PCL (**g**,**h**) 110 °C, (**i**,**j**) 130 °C, (**k**,**l**) 150 °C.
