# Supplementary material for: An Analysis of the Effect of Activation Temperature and Crack Geometry on the Healing Efficiency of Polycaprolactone (PCL)/Epoxy Blends
Source: Polymers (Basel). 2023 Jan 9;15(2):336. doi: 10.3390/polym15020336 (PMC9862102; doi:10.3390/polym15020336)
Supplement: Supplementary file 1 [file polymers-15-00336-s001.zip › polymers-2150972-supplementary.pdf]

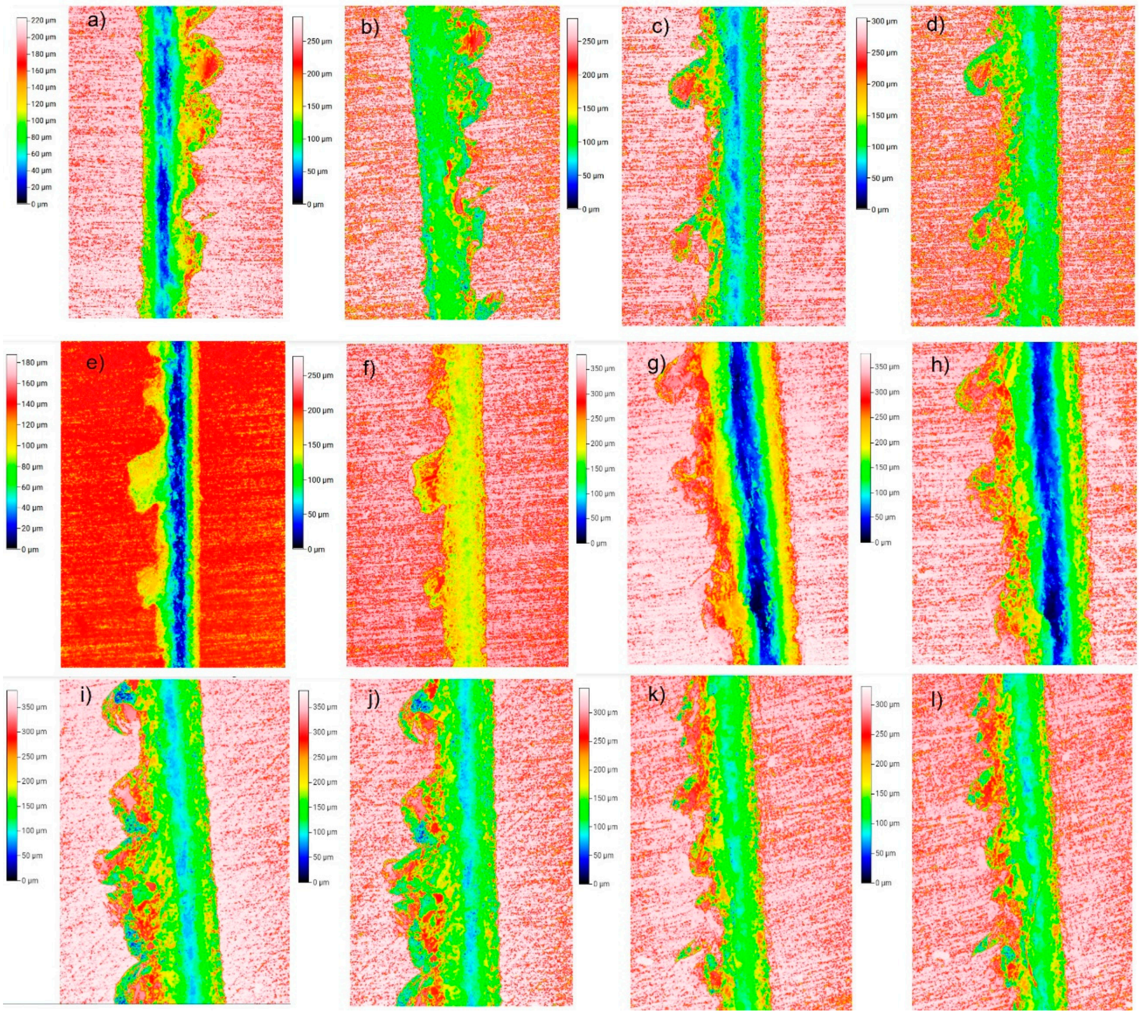

Figure S1. Images of optical perfilometry for PCL/epoxy blends with 10 % wt PCL before and after the self-healing process after15 minutes of heating at: (a), (b) 110 ºC , (c), (d) 130 ºC, (e), (f) 150 ºC and PCL/epoxy blends with 5 % wt PCL (g), (h) 110 ºC , (i), (j) 130 ºC, (k), (l) 150 ºC.
